# Transport of pyruvate into mitochondria is involved in methylmercury toxicity

**DOI:** 10.1038/srep21528

**Published:** 2016-02-22

**Authors:** Jin-Yong Lee, Yosuke Ishida, Tsutomu Takahashi, Akira Naganuma, Gi-Wook Hwang

**Affiliations:** 1 Laboratory of Molecular and Biochemical Toxicology, Graduate School of Pharmaceutical Sciences, Tohoku University, Sendai 980-8578, Japan; 2 Laboratory of Pharmaceutical Health Sciences, School of Pharmacy, Aichi Gakuin University, 1-100 Kusumoto-cho, Chikusa-ku, Nagoya 464-8650, Japan; 3 School of Pharmacy, Tokyo University of Pharmacy and Life Sciences, 1432-1 Horinouchi, Hachioji, Tokyo 192-0232, Japan

## Abstract

We have previously demonstrated that the overexpression of enzymes involved in the production of pyruvate, enolase 2 (Eno2) and *D*-lactate dehydrogenase (Dld3) renders yeast highly sensitive to methylmercury and that the promotion of intracellular pyruvate synthesis may be involved in intensifying the toxicity of methylmercury. In the present study, we showed that the addition of pyruvate to culture media in non-toxic concentrations significantly enhanced the sensitivity of yeast and human neuroblastoma cells to methylmercury. The results also suggested that methylmercury promoted the transport of pyruvate into mitochondria and that the increased pyruvate concentrations in mitochondria were involved in intensifying the toxicity of methylmercury without pyruvate being converted to acetyl-CoA. Furthermore, in human neuroblastoma cells, methylmercury treatment alone decreased the mitochondrial membrane potential, and the addition of pyruvate led to a further significant decrease. In addition, treatment with *N*-acetylcysteine (an antioxidant) significantly alleviated the toxicity of methylmercury and significantly inhibited the intensification of methylmercury toxicity by pyruvate. Based on these data, we hypothesize that methylmercury exerts its toxicity by raising the level of pyruvate in mitochondria and that mitochondrial dysfunction and increased levels of reactive oxygen species are involved in the action of pyruvate.

Minamata disease, which is characterized by disorders of the central nervous system, is widely known to be caused by environmental methylmercury pollution^1^,^2^. Methylmercury becomes highly concentrated in fish at higher levels of the food chain. In recent years, epidemiological investigations have shown that pregnant women who ingest relatively large amounts of methylmercury (e.g., through a fish-heavy diet) are at greater risk of giving birth to children with brain developmental disorders^3^,^4^. In response, the U.S. issued a national warning in 2001 recommending that pregnant women and infants limit their intake of fish, which was followed by similar warnings from Japan, the U.K., Canada, Australia and Norway. Thus, the influence of methylmercury on human health is a worldwide concern. The cellular mechanisms that cause methylmercury toxicity, however, continue to remain unclear, even some 50 years after the outbreak of Minamata disease.

We found that the ubiquitin-proteasome (UP) system, a protein degradation pathway, plays an important role as a defence mechanism against methylmercury toxicity^5^,^6^,^7^. Previous studies have suggested that some cellular proteins can intensify methylmercury toxicity and that this toxicity is decreased by the UP system, which promotes the degradation of these proteins. Recently, we have successfully identified proteins that are involved in intensifying methylmercury toxicity in yeast; the intracellular levels of these proteins are regulated by the UP system: Dld3, Grs1 and Eno2^8^. Of these three proteins, Dld3 is a *D*-lactate dehydrogenase involved in the conversion of *D*-lactose to pyruvate^9^, and Eno2 (enolase 2) is an enzyme involved in the conversion of glycerate 2-phosphate to phosphoenolpyruvate in the glycolytic pathway^10^. We observed that yeast exhibiting overexpression of Dld3 and Eno2, compared to wild-type yeast, were more sensitive to methylmercury, which suggests that an increase in intracellular pyruvate synthesis is somehow involved in intensifying the toxicity of methylmercury. Therefore, the present study sought to examine the role of pyruvate in methylmercury toxicity, using budding yeast as a model organism. Yeast has been established as a model organism in which powerful genetic approaches can be used to elucidate fundamental but complex eukaryotic processes^11^. Recently, yeast has been used as a model system to study the mechanisms of human neurodegenerative disorders^12^,^13^,^14^. Moreover, the similarities between yeast and human mitochondria facilitate the study of mitochondrial functions^15^. Examinations were also performed using IMR-32 cells, which are human-derived neuroblastoma cells.

## Results

### Pyruvate intensifies methylmercury toxicity in yeast

**Figure 1:**
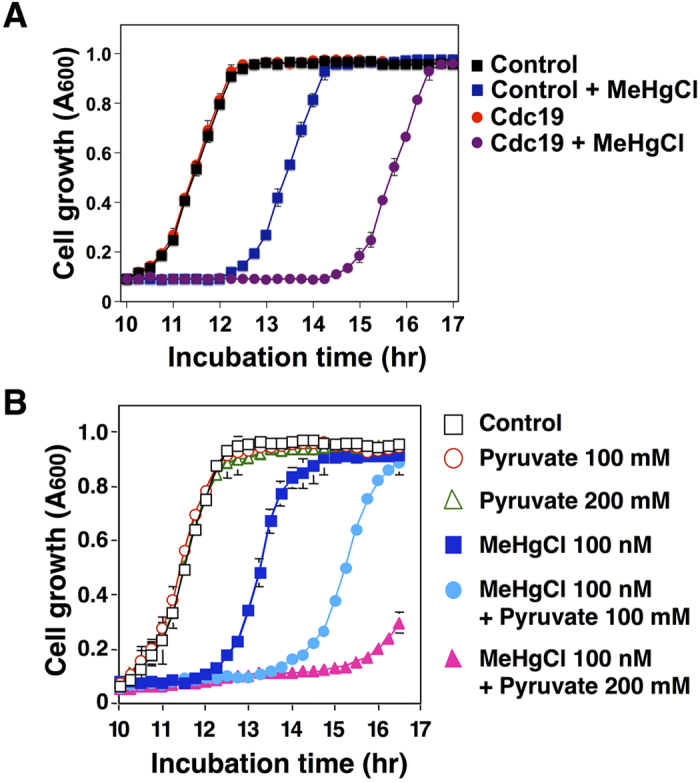
Pyruvate intensifies methylmercury toxicity in yeast. (**A**). Yeast cells (6.25 × 10^5^ cells/mL) expressing the indicated plasmids were cultured at 30 °C in SD liquid media containing methylmercuric chloride (MeHgCl; 100 nM). The absorbance was measured spectrophotometrically at 600 nm every 15 min for 24 hr. The data represent the mean ± S.D. of three cultures. The absence of a bracket indicates that the S.D. was within the area of the symbol. (**B**). Wild-type yeast cells (6.25 × 10^5^ cells/mL) were cultured at 30 °C in SD liquid medium containing methylmercuric chloride and/or sodium pyruvate at the indicated concentrations. The absorbance was measured spectrophotometrically at 600 nm every 15 min for 24 hr. The data represent the mean ± S.D. of three cultures. The absence of a bracket indicates that the S.D. was within the area of the symbol.

In the glycolytic system, glucose is converted to glycerate 2-phosphate in several stages and then to phosphoenolpyruvate; this conversion is mediated by Eno1 and Eno2. Phosphoenolpyruvate undergoes subsequent conversion to pyruvate, in a process mediated by Cdc19^16^. Because Cdc19 acts downstream of Eno2, we examined how the overexpression of Cdc19 could potentially influence the sensitivity of yeast to methylmercury. In this approach, yeast cells that overexpressed Cdc19 demonstrated a high sensitivity to methylmercury (Fig. 1A). When non-toxic concentrations of pyruvate were added to the culture media, yeast growth was more intensively inhibited by methylmercury and was dependent on the concentration of pyruvate (Fig. 1B). These results suggest that an increase in pyruvate levels in yeast is involved in intensifying the toxicity of methylmercury.

### Methylmercury toxicity is intensified by promoting the transport of pyruvate into mitochondria

**Figure 2:**
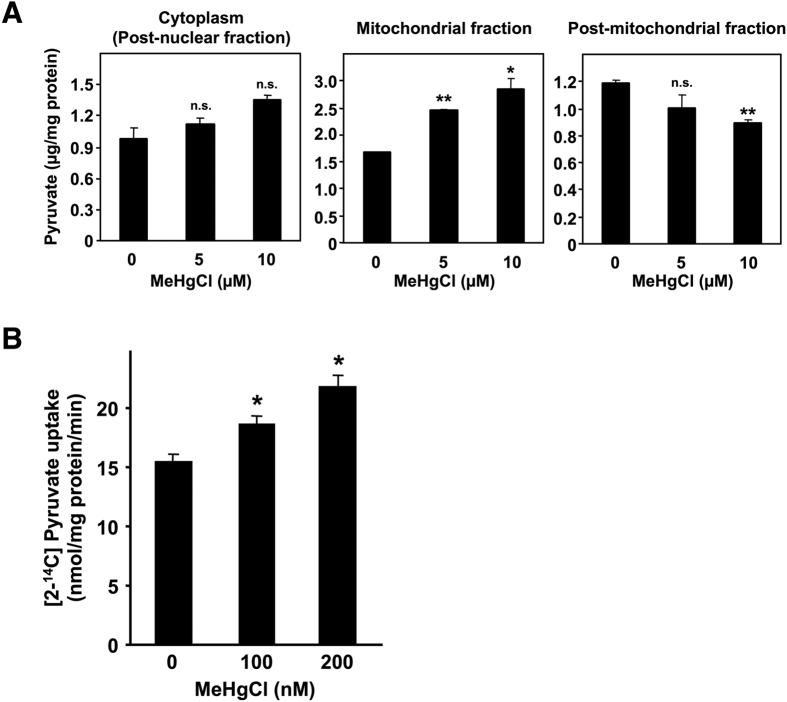
Methylmercury induces the transport of pyruvate into the mitochondria in yeast. (**A**). Spheroplasts (10^8^ cells/mL) were incubated with gentle shaking at 30 °C in sorbitol-containing SD liquid medium that also contained methylmercuric chloride at the indicated concentrations. After a 1 hr incubation, post-nuclear, mitochondrial and post-mitochondrial fractions were prepared at 4 °C. The pyruvate levels of each fraction were measured by β-NADH consumption using pyruvate reduction at 340 nm. Significant differences were observed relative to the control group without methylmercury treatment. n.s.: no significance, *p < 0.05, **p < 0.01 (**B**). The uptake of ^14^C-labelled pyruvate into intact mitochondria was measured after treatment with methylmercuric acid *in vitro*. The imported pyruvate was quantified using a liquid scintillation counter, and normalized to the amount of mitochondrial protein. Significant differences were observed relative to the control group (without methylmercury treatment). *p < 0.01.

Pyruvate is produced in the cytoplasm and is then transported into the mitochondria, where it is converted to acetyl-CoA; acetyl-CoA subsequently reacts with oxaloacetate to form citrate, thus entering the tricarboxylic acid (TCA) cycle. Following the suggestion that pyruvate is involved in methylmercury toxicity, we examined the influence of methylmercury on the level and distribution of pyruvate in yeast. Because spheroplasting during cell fractionation may alter the metabolism or distribution of pyruvate, in this study we spheroplasted yeast cells prior to treatment with methylmercury. Moreover, we used a large quantity of spheroplasts to measure the pyruvate levels because endogenous pyruvate levels can be measured by using a large quantity of spheroplasts. The accuracy of cell fractionation was confirmed via Western blotting, using antibodies against cytochrome c oxidase subunit III, a mitochondrial protein marker, or 3-phosphoglycerate kinase, a cytoplasmic protein marker (Supplementary Figure 1). In this study, pyruvate levels in the cytoplasmic fraction (obtained by removing nuclei from the homogenate) tended to increase after exposure to methylmercury, although the change was not statistically significant (Fig. 2A). In addition, pyruvate levels in the mitochondria exhibited a significant increase depending on the concentration of added methylmercury, and pyruvate levels in the post-mitochondrial fraction (obtained by removing mitochondria from the cytoplasm) in contrast decreased, depending on the concentration of methylmercury (Fig. 2A). In addition, the cytotoxicity caused by methylmercury under the present set of conditions was 10% or less (data not shown). Measurement of the transport of radioactive pyruvate into isolated mitochondria showed increased transportation of pyruvate into the mitochondria and was dependent on the concentration of methylmercury used during the treatment (Fig. 2B). Based on these observations, we hypothesize that methylmercury promotes the transport of pyruvate into mitochondria.

**Figure 3:**
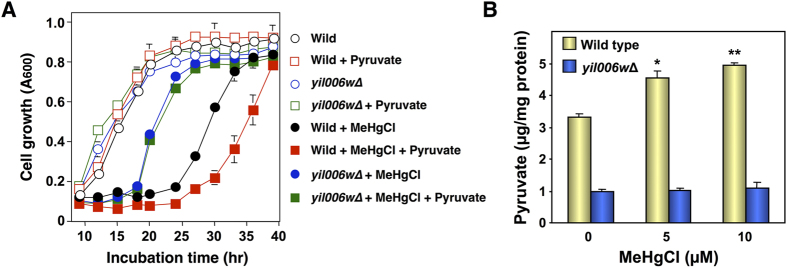
Yil006w is involved in the methylmercury-mediated transport of pyruvate into mitochondria in yeast. (**A**). Yeast cells were cultured at 30 °C in SD liquid medium containing methylmercuric chloride and/or sodium pyruvate at the indicated concentrations. The absorbance at 600 nm was measured using a spectrophotometer every 3 hr for 48 hr. The data represent the mean ± S.D. of three cultures. The absence of a bracket indicates that the S.D. was within the area of the symbol. (**B)**. Spheroplasts were incubated with gentle shaking at 30 °C in sorbitol-SD medium containing methylmercuric chloride at the indicated concentrations. After a 1 hr incubation, the mitochondrial fraction was prepared at 4 °C. Pyruvate levels were measured by β-NADH consumption using pyruvate reduction at 34 nm. Significant differences were observed compared to the control group without methylmercury treatment. *p < 0.05, **p < 0.01.

Pyruvate synthesized in the cytoplasm of yeast is transported into the mitochondrial matrix, a process mediated by the Yil006w transporter that is present in the inner mitochondrial membrane^17^. We therefore examined the sensitivity of Yil006w-deleted yeast to methylmercury to determine the relationship between methylmercury toxicity and pyruvate transport into mitochondria. In this approach, Yil006w-deleted yeast (*yil006w*Δ) demonstrated an enhanced resistance to methylmercury compared to that of wild-type yeast (Fig. 3A). In addition, in Yil006w-deleted yeast the toxicity of methylmercury was only minimally intensified by pyruvate (Fig. 3A), and pyruvate levels in the mitochondria were lower and were not increased after treatment with methylmercury (Fig. 3B), in contrast to wild-type yeast. Based on these observations, we hypothesize that methylmercury toxicity is intensified by promoting the transport of pyruvate into mitochondria, a process mediated by Yil006w.

### Pyruvate is involved in intensifying methylmercury toxicity without being metabolized to acetyl-CoA

**Figure 4:**
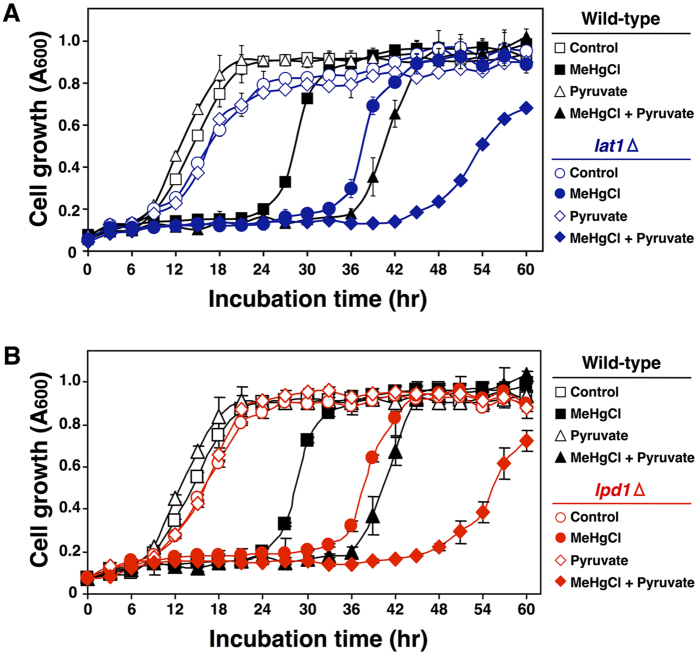
Pyruvate is not converted to acetyl-CoA in the mitochondria matrix and is involved in methylmercury toxicity. Yeast cells with deletions of the indicated gene(s) ((**A)** LAT1, (**B**) LPD1) were cultured at 30 °C in SD liquid medium containing methylmercuric chloride and/or sodium pyruvate at the indicated concentrations. The absorbance was measured with a spectrophotometer at 600 nm every 3 hr for 48 hr. The data represent the mean ± S.D. of three cultures. The absence of a bracket indicates that the S.D. was within the area of the symbol.

Pyruvate that is transported into mitochondria is converted to acetyl-CoA before entering the TCA cycle and is thus involved in ATP production. Pyruvate dehydrogenase is an enzyme that converts pyruvate to acetyl-CoA and is composed of several proteins: Pda1, Pdb1, Lat1, Lpd1 and Pdx1^18^. To determine the relationship between methylmercury toxicity and the conversion of pyruvate to acetyl-CoA, we examined the influence of the removal of pyruvate dehydrogenase components Pda1, Pdb1, Lat1 and Lpd1. In this approach, yeast with deletions of the respective components, compared to wild-type yeast, consistently exhibited a higher sensitivity to methylmercury (data not shown). We subsequently examined how the addition of pyruvate to the culture media influenced methylmercury toxicity using yeast cells with Lat1 deleted (dihydrolipoyl transacetylase, the active centre of pyruvate dehydrogenase)^19^,^20^. In this approach, Lat1-deleted yeast (*lat1*Δ) which are almost completely lack pyruvate dehydrogenase activity were more sensitive to methylmercury than were wild-type yeast and were more sensitive to methylmercury toxicity by pyruvate (Fig. 4A). Similar results were obtained with Lpd1-deleted yeast (*lpd1*Δ) (Fig. 4B). Based on these results, we hypothesize that pyruvate that is transported into mitochondria is involved in intensifying methylmercury toxicity without being converted to acetyl-CoA.

### Methylmercury causes mitochondrial dysfunction by promoting the transport of pyruvate into mitochondria in human neuroblastoma cells

**Figure 5:**
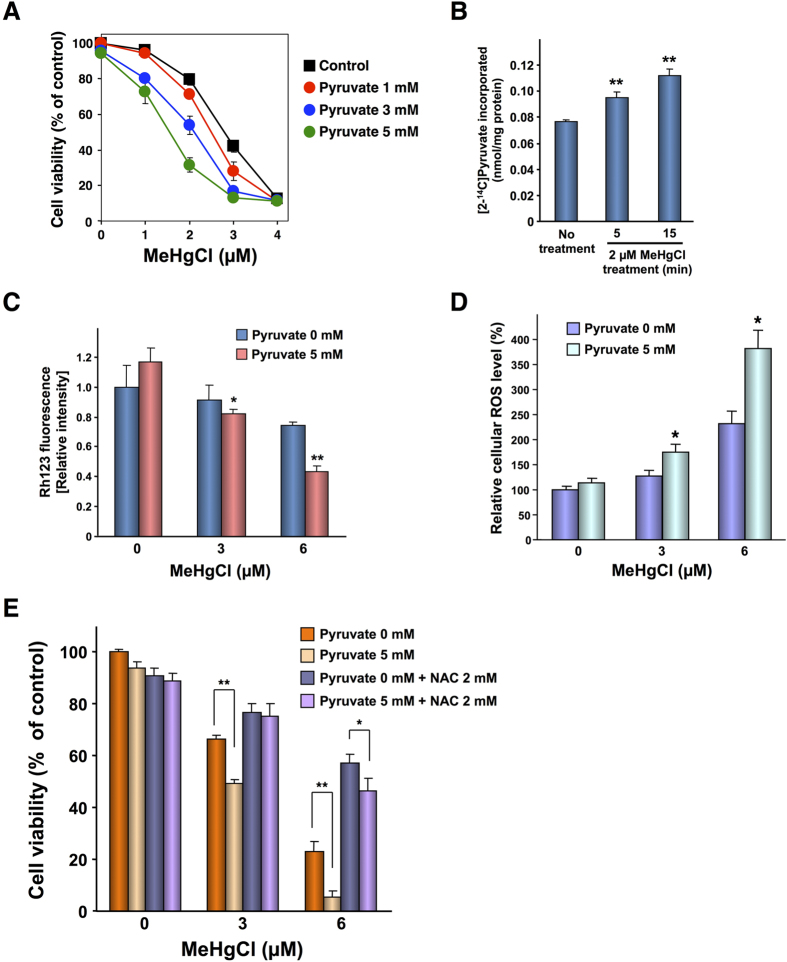
Methylmercury causes mitochondrial dysfunction by promoting the uptake of pyruvate into the mitochondria in IMR-32 cells. (**A**). Cell viability was measured using an Alamar blue solution. The data represent the mean ± S.D. of three cultures. The absence of a bracket indicates that the S.D. was within the area of the symbol. (**B**). [2-^14^C] Pyruvate levels in mitochondria were measured using a liquid scintillation counter. Significant differences were observed compared to the control group without pyruvate treatment. *p < 0.05, **p < 0.01 (**C**). Rhodamine 123 levels were measured at an excitation wavelength of 488 nm and an emission wavelength of 530 nm. Significant differences were observed relative to the control group without pyruvate treatment. *p < 0.05, **p < 0.01 (**D**). IMR-32 cells (2 × 10^4^ cells/80 μL) were seeded into each well of a 96-well plate. After incubation for 24 hr, 10 μM H_2_DCF-DA was added to each well. One hour later, the cells were treated with methylmercuric chloride and/or sodium pyruvate and allowed to remain for 6 hr in the dark. After the treatment, the media were removed, and the cells were washed with HBSS. After washing, 100 μL HBSS was added to each well and the fluorescence was measured at an excitation wavelength of 485 nm and an emission wavelength of 530 nm. Subsequently, the HBSS was replaced with DMEM containing 10% Alamar blue solution. The cell viability was measured at an excitation wavelength of 544 nm and an emission wavelength of 590 nm. Susceptibility was calculated as ROS level/viability and is presented as a percentage of non-treated cells. Data represent the mean ± S.D. (brackets) of six cultures. Significant differences were observed relative to the control group without pyruvate treatment. *p < 0.01 (**E**). Cell viability was measured using an Alamar blue solution. The data represent the mean ± S.D. (brackets) of three cultures. Significant differences were observed relative to the control group without pyruvate treatment. *p < 0.05, **p < 0.01.

Our observations in yeast suggested that methylmercury produces cytotoxicity by increasing the pyruvate levels in mitochondria. We next investigated the influence of pyruvate on methylmercury toxicity; we used human neuroblastoma cells (IMR-32) because methylmercury is a neurotoxic substance. In this approach, using an Alamar blue assay (Fig. 5A) and an MTT assay (Supplementary Figure 2), we observed that the addition of non-toxic concentrations of pyruvate to the culture media significantly enhanced the sensitivity of the cells to methylmercury, a result similar to the response observed in yeast. We next examined the influence of methylmercury on pyruvate levels in the mitochondria of IMR-32 cells. However, it is very difficult to measure the levels of endogenous pyruvate in human cultured cells. Therefore, IMR-32 cells were treated with radioactive pyruvate for 90 min, followed by treatment with methylmercury for up to 15 min, before the level of radioactive pyruvate in mitochondrial fractions was measured with a liquid scintillator. In this approach, a brief treatment with methylmercury significantly increased the level of radioactive pyruvate in the mitochondria (Fig. 5B). In addition, the effectiveness of cell fractionation was confirmed via western blotting, using antibodies against cytochrome c oxidase subunit IV, a mitochondrial marker protein, or glyceraldehyde 3-phosphate dehydrogenase, a cytoplasmic protein marker (Supplementary Figure 3). These results suggested that methylmercury causes cellular disorders by promoting the transport of pyruvate into mitochondria in human-derived IMR-32 cells as well as in yeast.

Mitochondria are organelles that produce energy by promoting membrane electrogenesis, due to a pH or potential difference between the outer side and matrix side of the inner membrane. If the membrane potential decreases, low-molecular-weight substances (including protons) flow into the mitochondria, causing mitochondrial dysfunction^21^,^22^. The results shown in Fig. 4 suggested that pyruvate that is transported into mitochondria is involved in intensifying methylmercury toxicity without being converted to acetyl-CoA. There is a possibility that pyruvate transported into mitochondria influences the mitochondrial membrane potential as an organic acid. We therefore investigated the relationship between the mitochondrial membrane potential and the intensification of methylmercury toxicity mediated by pyruvate. The membrane potential was measured using rhodamine 123, a cationic fluorescent substance that accumulates in response to an increase of anions on the matrix side of the mitochondria^23^. In this approach, treatment with methylmercury alone for 3 hr decreased the mitochondrial membrane potential, and the addition of pyruvate resulted in an additional, significant decrease (Fig. 5C). This result suggests that mitochondrial dysfunction is involved in the intensification of methylmercury toxicity mediated by pyruvate. In addition, methylmercury is known to promote the production of reactive oxygen species in mitochondria^24^,^25^,^26^,^27^,^28^. We therefore investigated the relationship between reactive oxygen species and the intensification of methylmercury toxicity mediated by pyruvate. In this approach, treatment with methylmercury alone for 6 hr increased the intracellular levels of reactive oxygen species, and the addition of pyruvate further increased the production of reactive oxygen species, depending on the concentration of pyruvate used (Fig. 5D). However, treatment with pyruvate alone did not influence the intracellular levels of reactive oxygen species. In addition, the levels of reactive oxygen species were not changed after treatment for 3 hr with methylmercury alone or in combination with pyruvate (data not shown). These results suggest that the production of reactive oxygen species via the reduction of the mitochondrial membrane potential may be involved in the intensification of methylmercury toxicity by pyruvate. Next, we examined the influence of *N*-acetylcysteine (NAC), an antioxidant, on the intensification of methylmercury toxicity mediated by pyruvate. NAC is a cysteine derivative that may bind to methylmercury in culture media. Therefore, we treated IMR-32 cells with NAC for 6 hr and subsequently replaced the culture media to remove NAC prior to treatment with methylmercury or pyruvate. In this approach, treatment with NAC significantly alleviated methylmercury toxicity and significantly inhibited the intensification of methylmercury toxicity mediated by pyruvate (Fig. 5F). In addition, the cell viability was not changed after treatment with methylmercury alone or in combination with pyruvate for 6 hr (data not shown). These results strongly suggest that reactive oxygen species are involved in the intensification of methylmercury toxicity mediated by pyruvate.

## Discussion

Pyruvate produced from glycolysis is involved in the production of ATP and in homeostasis of carbohydrates, fats and amino acids^29^,^30^. It has also been reported that some cells release pyruvate into blood plasma and serum, and the released pyruvate reacts with extracellular H_2_O_2_ independently of enzymes to produce an antioxidative action^31^,^32^. Interestingly, our study found that excessive pyruvate transported into mitochondria intensified the toxicity of methylmercury without being metabolized to acetyl-CoA.

Excessive pyruvate added to culture media did not change pyruvate levels in mitochondria and did not lead to cytotoxicity (data not shown), indicating that the normal level of pyruvate in mitochondria is under strict control. However, in the presence of methylmercury, we observed enhanced transport of pyruvate into mitochondria. Furthermore, the deletion of the Yil006w transporter (which is involved in transporting pyruvate into mitochondria) did not result in any intensification of pyruvate-mediated methylmercury toxicity. Based on these observations, we can hypothesize that the toxicity of methylmercury is intensified by increasing the pyruvate levels in mitochondria. Normally, pyruvate is involved in the production of ATP via the TCA cycle, following its transportation into mitochondria. However, deletion of pyruvate dehydrogenase, which catalyses the metabolism of pyruvate, resulted in a further intensification of pyruvate-mediated methylmercury toxicity, suggesting that pyruvate intensifies the toxicity of methylmercury without being metabolized in the TCA cycle.

However, it cannot be excluded that pyruvate dehydrogenase is somehow involved in the pyruvate-mediated intensification of methylmercury toxicity. Pyruvate dehydrogenase, which converts pyruvate to acetyl-CoA, is a complex composed of three enzymes, and the primary active site is comprised of lipoic acid bound to a Lys residue of dihydrolipoyl transacetylase (Lat1)^33^. This lipoic acid has sulfhydryl groups that form a disulphide bond in a reversible manner, and the resulting redox reaction decarboxylates pyruvate and transfers an acetyl group to the decarboxylated pyruvate. Methylmercury is known to bind strongly to sulfhydryl groups and block the activity of sulfhydryl enzymes^34^. This leaves the possibility that methylmercury transported into mitochondria binds to the sulfhydryl groups of Lat1 and thus blocks the activity of pyruvate dehydrogenase, resulting in accumulation of pyruvate within the mitochondria. Therefore, we examined the influence of methylmercury on the activity of pyruvate dehydrogenase. In this approach, treatment with methylmercury caused only an ~10% decrease in the activity of pyruvate dehydrogenase, a small difference compared to the control in yeast and IMR-32 cells (data not shown). We can thus hypothesize that blockage of pyruvate dehydrogenase activity by methylmercury is essentially not involved when methylmercury increases the pyruvate levels in mitochondria.

Some authors, in examining mitochondria isolated from rat liver cells, have suggested that methylmercury opens a mitochondrial membrane permeability transition pore and promotes influx of anions such as nitrate, thus inducing mitochondrial swelling and leading to cell death^35^. Previous studies have indicated that pyruvate strengthens the dihydrolipoate-induced mitochondrial permeability transition and mitochondrial swelling in isolated rat liver mitochondria^36^. The increase in mitochondrial permeability has been reported to be involved in neuronal injury^37^, and mitochondrial swelling has been found to be induced in the cortices in an Alzheimer disease mouse model^38^. Therefore, the disruption of the mitochondrial permeability transition and of mitochondrial swelling may be involved in the mechanism of pyruvate-mediated methylmercury toxicity. It is possible that methylmercury has some influence on transporters that are present in mitochondria, such as Yil006w, and hence promotes the transport of pyruvate into mitochondria. The results in Fig. 3B show that pyruvate levels in mitochondria from Yil006-deleted yeast were approximately 30% of those in wild-type yeast, indicating that mitochondria also have another mechanism involved in the transport of pyruvate in addition to Yil006w. However, transport mechanisms other than Yil006w do not appear to be involved when methylmercury promotes the transport of pyruvate into mitochondria, given that methylmercury did not increase pyruvate concentrations in mitochondria following the deletion of Yil006w.

One study has reported that methylmercury exhibits cytotoxicity by increasing mitochondrial membrane permeability, thus increasing the release of calcium from mitochondria to the cytoplasm^39^,^40^. Other reports have raised the possibility that methylmercury promotes the production of reactive oxygen species by inhibiting the activity of complex III of the mitochondrial electron transport chain^41^ and that methylmercury blocks production of ATP by inhibiting the function of complex IV^42^. Thus, mitochondria can be regarded as intracellular targets for methylmercury. The addition of pyruvate minimally influenced the cytotoxicity of cadmium, arsenic trioxide and other substances (data not shown), which suggests that the intensification of toxicity mediated by pyruvate may be specific to methylmercury toxicity.

Our present findings suggest the existence of a new mechanism for the toxicity of methylmercury that targets mitochondria, in addition to the above-mentioned known toxic mechanism of methylmercury acting through mitochondria. The mechanisms related to methylmercury toxicity may be clarified by studying in detail the relationship between the level of pyruvate in mitochondria and the known toxic mechanism of methylmercury acting via mitochondria.

## Materials and Methods

### Yeast strain and growth culture conditions

*Saccharomyces cerevisiae* BY4742 (*MATα his3*Δ*1 leu2*Δ*0 lys2*Δ*0 ura3*Δ*0*) and deletion (knock-out) strains constructed by insertion of kanMX4 cassettes (conferring G418 resistance as a selective marker of the BY4742 genome) were obtained from Euroscarf (Frankfurt, Germany). Yeast cells were grown in yeast extract-peptone-dextrose (YPD) medium or synthetic dextrose (SD) medium at 30 °C. Plasmid DNA was introduced into BY4742 cells using the high-efficiency lithium acetate transformation method^43^.

### Yeast growth curve

Yeast cells (6.25 × 10^5^ cells/mL) were grown in synthetic dextrose (SD) medium containing methylmercuric chloride and sodium pyruvate at the indicated concentrations in 96-well plates. The yeast cells were incubated for 48 hr at 30 °C with shaking. The absorbance of each culture at 600 nm was measured every 3 hr to quantify cell growth.

### Spheroplasting and fractionation

Spheroplasts and mitochondria were prepared according to previously published methods^44^. Yeast cells were grown in 1 L of SD liquid medium until the optical density at 600 nm was approximately 1.7 (10^11^ cells/L). The yeast cells were collected and incubated in 60 mL of Tris buffer (0.1 M Tris-SO_4_, pH 9.4, 10 mM DTT) for 10 min at 30 °C with gentle shaking (100 rpm, Bio-shaker BR-40LF, Taitec, Saitama, Japan). After the yeast cells were centrifuged at 2,000 × g for 5 min, the pellet was washed with 60 mL of spheroplasting buffer (1.2 M sorbitol, 20 mM KPi, pH 7.4), collected by centrifugation (2,000 × g for 5 min) and then suspended in 60 mL of spheroplasting buffer. For digesting yeast cell walls, the suspension was incubated with zymolyase (2 mg zymolyase/g yeast) for 15 to 30 min at 30 °C with gentle shaking (100 rpm, Bio-shaker BR-40LF, Taitec). To confirm spheroplasting, a small aliquot (20 μL) of yeast suspension was added to 1 mL of distilled water. When the yeast cells appeared to be collapsed due to the osmotic pressure, they were considered to be completely spheroplasted.

The spheroplasts (2 × 10^10^ cells) were suspended in 200 mL of SD medium containing 1.2 M sorbitol to an optical density of 0.85 at 600 nm, and treated with methylmercuric chloride for 1 hr at 30 °C with gentle shaking (100 rpm, Bio-shaker BR-40LF, Taitec). Methylmercury-treated spheroplasts were washed twice with ice-cold spheroplasting buffer, suspended in 3 mL of mitochondrial isolation buffer (MIB) (0.6 M sorbitol, 20 mM HEPES-KOH, pH 7.4, 1 mM PMSF, 0.5 mM EDTA), and then homogenized on ice with a Dounce homogenizer (Wheaton, Millville, NJ, USA) and 20 strokes with a tight-fitting pestle. The homogenate was divided into mitochondrial, post-mitochondrial and post-nuclear fractions.

For the mitochondrial fraction, part of the homogenate was centrifuged at 1,000 × g for 5 min. The supernatant was transferred to another tube, and the pellet was resuspended in 2 mL of MIB followed by homogenization and centrifugation at 1,500 × g for 5 min. The supernatant was combined with the previously prepared supernatant. The supernatant was centrifuged at 12,000 × g for 10 min, and the pellet was suspended in 10 mL of MIB. After centrifugation at 1,500 × g for 5 min, the supernatant was collected and centrifuged at 12,000 × g for 10 min, and the pellet was suspended in MIB without phenylmethylsulfonyl fluoride. The suspension was centrifuged at 12,000 × g for 10 min, and the pellet (the mitochondrial fraction) was suspended in 1 mL of PBS.

For the post-mitochondrial fraction, the homogenate was centrifuged at 20,000 × g for 30 min, and this supernatant was used as the post-mitochondrial fraction. For the post-nuclear fraction, the homogenate was centrifuged at 1,500 × g for 30 min. This supernatant was used as the post-nuclear fraction.

### Quantification of pyruvate

The protein concentration from each fraction was determined using a DC protein assay (Bio-Rad, Hercules, CA, USA), and a 1 mL fraction was used for the quantification of pyruvate. Each fraction was lysed by sonication for 30 sec (output 4, duty 30%, Branson Sonifier 450). The lysate was filtered via centrifugation (8,000 × g) for 10 min using a 0.1 μm pore micro-filter. The filtrate was incubated with 500 μM β-NADH and 2 units of *L*-lactate dehydrogenase in 0.5 M Tris buffer, pH 7.4, at 37 °C for 30 sec. The consumption of β-NADH was determined using the absorbance at 340 nm to represent the amount of pyruvate^45^.

### IMR-32 cell culture and viability

Human neuroblastoma (IMR-32 cells) were cultured in Dulbecco’s Modified Eagle’s Medium (DMEM) (Sigma, St. Louis, MO, USA) supplemented with 10% (v/v) foetal bovine serum (Bio-west, Kansas City, MO, USA), 0.06% L-glutamine, and 100 U/mL penicillin (Invitrogen, Grand Island, NY, USA) at 37 °C in a humidified incubator containing 5% (v/v) CO_2_. IMR-32 cells (2 × 10^4^ cells/well) were seeded into 96-well plates for 24 hr and treated with methylmercuric chloride and/or pyruvate at the indicated concentrations for 24 hr. Cell viability was measured using a 10% Alamar blue solution using an excitation wavelength of 544 nm and an emission wavelength of 590 nm.

### Crude mitochondrial isolation from IMR-32 cells

Crude mitochondria were isolated according to previously published methods^46^. The cells were suspended in mitochondrial isolation buffer (10 mM NaCl, 1.5 mM CaCl_2_, 10 mM Tris-HCl, pH 7.5) and incubated on ice for 10 min. After the incubation, the cell suspension was homogenized using 30 strokes with a Dounce homogenizer with a tight-fitting pestle on ice. The homogenate was centrifuged at 600 × g for 10 min, followed by centrifugation of the supernatant at 6,000 × g for 10 min. The pellet was washed twice with mitochondrial isolation buffer then suspended in PBS and used as the crude mitochondrial fraction.

### Pyruvate incorporation into mitochondria in IMR-32 cells

IMR-32 cells (6 × 10^6^ cells) were seeded into 15 cm culture dishes in 30 mL pyruvate-free DMEM and cultured for 24 hr. The cells were treated with 0.05 μCi/mL of [2-^14^C] pyruvate (PerkinElmer Inc., Waltham, MA, USA) for 90 min followed by addition of methylmercuric chloride to the cell culture. After incubation, the mitochondria were isolated from the cells. The amount of [2-^14^C] pyruvate incorporated into the mitochondria was measured using a liquid scintillation counter and was normalized to the amount of mitochondrial protein^47^.

### Mitochondrial membrane potential

IMR-32 cells were seeded into 6-well plates at a concentration of 4 × 10^5^ cells/2 mL/well. After a 24 hr cultivation period, the indicated concentrations of methylmercuric chloride and/or pyruvate were added, followed by incubation for 3 hr. After incubation and washing with Hank’s Balanced Salt Solution (HBSS) buffer, the cells were incubated with 5 μM rhodamine 123 (Sigma) in HBSS buffer for 30 min in the dark. Rhodamine 123-treated cells were collected with 600 μL of ice-cold HBSS buffer. To measure the intensity of rhodamine 123, 100 μL of cell suspension was transferred to half of the wells of a 96-well plate, and the fluorescence was measured at an excitation wavelength of 488 nm and an emission wavelength of 530 nm. Cells in 300 μL suspensions were counted for the normalization of the mitochondrial membrane potential^48^,^49^.

## Additional Information

**How to cite this article**: Lee, J.-Y. *et al.* Transport of pyruvate into mitochondria is involved in methylmercury toxicity. *Sci. Rep.* **6**, 21528; doi: 10.1038/srep21528 (2016).

## Supplementary Material

Supplementary Figures
