# Supplementary material for: Transport of pyruvate into mitochondria is involved in methylmercury toxicity
Source: Sci Rep. 2016 Feb 22;6:21528. doi: 10.1038/srep21528 (PMC4761912; doi:10.1038/srep21528)
Supplement: Supplementary Figures [file srep21528-s1.doc]

**Supplementary Figures**

**Transport of pyruvate into mitochondria is involved in methylmercury toxicity**

Jin-Yong Lee^a, b^, Yosuke Ishida^a^, Tsutomu Takahashi^a, c^, Akira Naganuma^a^ and Gi-Wook Hwang^a^, *

^a^ Laboratory of Molecular and Biochemical Toxicology, Graduate School of Pharmaceutical Sciences, Tohoku University, Sendai 980-8578, Japan

^b^ Laboratory of Pharmaceutical Health Sciences, School of Pharmacy, Aichi Gakuin University, 1-100 Kusumoto-cho, Chikusa-ku, Nagoya 464-8650, Japan

^c^ School of Pharmacy, Tokyo University of Pharmacy and Life Sciences, 1432-1 Horinouchi, Hachioji, Tokyo 192-0232, Japan

------------------------------------

*Address for all correspondence:

Gi-Wook Hwang, Ph.D.

Laboratory of Molecular and Biochemical Toxicology, Graduate School of Pharmaceutical Sciences, Tohoku University, Sendai 980-8578, Japan

Phone & Fax: +81-22-795-6872

E-mail: gwhwang@m.tohoku.ac.jp

**Supplementary Figure 1**. Validation of fractionation in yeast. Each fraction was subjected to immunoblotting analysis with indicated antibodies.

**Supplementary Figure 2**. The viability of IMR-32 cells after methylmercury treatment using the MTT assay. IMR-32 cells (2×10^4^ cells/well) were seeded onto 96-well plates for 24 hr and treated with methylmercuric chloride and/or pyruvate at the indicated concentrations for 24 hr. After treatment, culture medium was changed to fresh 10% fetal bovine serum – DMEM containing 0.5 mg/mL MTT [3-(4,5-Dimethyl-2-thiazolyl)-2,5-diphenyl-*2H*-tetrazolium bromide] and incubated for another 4 hr at 37°C. After removing the media, dimethyl sulfoxide was added to MTT formazan. Absorbance at 570 nm was measured using a microplate spectrophotometer. Data represent the mean S.D. of three cultures. The absence of a bracket indicates that the S.D. was within the area of the symbol.

**Supplementary Figure 3**. Validation of fractionation in IMR-32 cells. Each fraction was subjected to immunoblotting analysis with indicated antibodies.
